# Enhancing Standardized and Structured Recording by Elderly Care Physicians for Reusing Electronic Health Record Data: Interview Study

**DOI:** 10.2196/63710

**Published:** 2024-12-13

**Authors:** Charlotte A W Albers, Yvonne Wieland-Jorna, Martine C de Bruijne, Martin Smalbrugge, Karlijn J Joling, Marike E de Boer

**Affiliations:** 1 Department of Medicine for Older People Location Vrije Universiteit Amsterdam Amsterdam UMC Amsterdam Netherlands; 2 Aging & Later Life Research Program Amsterdam Public Health Amsterdam Netherlands; 3 Methodology Amsterdam Public Health Research Program Amsterdam Public Health Amsterdam Netherlands; 4 Netherlands Institute for Health Services Research Utrecht Netherlands; 5 Department of Public and Occupational Health Location Vrije Universiteit Amsterdam Amsterdam UMC Amsterdam Netherlands; 6 Quality of Care Research Program Amsterdam Public Health Amsterdam Netherlands

**Keywords:** electronic health records, health information interoperability, health information exchange, reference standards, long-term care, nursing homes, medical records, attitude of health personnel, qualitative research, digital health

## Abstract

**Background:**

Elderly care physicians (ECPs) in nursing homes document patients’ health, medical conditions, and the care provided in electronic health records (EHRs). However, much of these health data currently lack structure and standardization, limiting their potential for health information exchange across care providers and reuse for quality improvement, policy development, and scientific research. Enhancing this potential requires insight into the attitudes and behaviors of ECPs toward standardized and structured recording in EHRs.

**Objective:**

This study aims to answer why and how ECPs record their findings in EHRs and what factors influence them to record in a standardized and structured manner. The findings will be used to formulate recommendations aimed at enhancing standardized and structured data recording for the reuse of EHR data.

**Methods:**

Semistructured interviews were conducted with 13 ECPs working in Dutch nursing homes. We recruited participants through purposive sampling, aiming for diversity in age, gender, health care organization, and use of EHR systems. Interviews continued until we reached data saturation. Analysis was performed using inductive thematic analysis.

**Results:**

ECPs primarily use EHRs to document daily patient care, ensure continuity of care, and fulfill their obligation to record specific information for accountability purposes. The EHR serves as a record to justify their actions in the event of a complaint. In addition, some respondents also mentioned recording information for secondary purposes, such as research and quality improvement. Several factors were found to influence standardized and structured recording. At a personal level, it is crucial to experience the added value of standardized and structured recording. At the organizational level, clear internal guidelines and a focus on their implementation can have a substantial impact. At the level of the EHR system, user-friendliness, interoperability, and guidance were most frequently mentioned as being important. At a national level, the alignment of internal guidelines with overarching standards plays a pivotal role in encouraging standardized and structured recording.

**Conclusions:**

The results of our study are similar to the findings of previous research in hospital care and general practice. Therefore, long-term care can learn from solutions regarding standardized and structured recording in other health care sectors. The main motives for ECPs to record in EHRs are the daily patient care and ensuring continuity of care. Standardized and structured recording can be improved by aligning the recording method in EHRs with the primary care process. In addition, there are incentives for motivating ECPs to record in a standardized and structured way, mainly at the personal, organizational, EHR system, and national levels.

## Introduction

### Background

Since the introduction of the first electronic medical record in 1972 [1] and its evolution into an electronic health record (EHR) in the subsequent years, there have been substantial changes in how and why health care professionals use these records. Initially, they served as a digital notebook for physicians to record their thoughts and actions related to patients’ treatment. Today, EHRs have evolved into a multipurpose data source. Primary use of health data supports direct patient care, and secondary use refers to its application for other purposes, such as scientific and clinical research [2-4], quality improvement [5,6], and policy development [7]. The ability of electronic health systems to exchange and use information across health care organizations refers to interoperability [8,9]. Effective interoperability is essential for both primary and secondary use of data and requires high-quality data to ensure that the data shared across health care organizations and their electronic systems retain their meaning (semantic interoperability) [10].

Standardized and structured recording of patient information is crucial for ensuring interoperability across electronic health systems and supporting both primary and secondary uses of high-quality health data [11-13]. Standardized recording refers to the transfer of data from various formats into a common format [14]. This might involve the use of a medical coding classification system such as the *International Classification of Diseases* (*ICD*) [15] or the *International Classification of Primary Care* (*ICPC*) [16]. Terminology systems such as Systematized Nomenclature of Medicine Clinical Terms (SNOMED CT) and Logical Observation, Identifiers, Names and Codes (LOINC) can be supportive by cross-mapping to other international terminologies, classifications, and code systems [17] to further increase semantic interoperability [18-20]. Coding and terminology systems allow health care professionals to record patient information in a standardized way, ensuring that the same clinical concepts are consistently represented across different systems. Structured recording is defined as allocating specific fields within EHRs to store certain health data, such as diagnoses and blood pressure [21].

In some health care settings, such as general practice and hospital care, steps have been taken to work toward standardized and structured recording of patient information. Coding systems, such as *ICD*-10/11 and *ICPC*, are increasingly being used by health care professionals [22-24]. In practice, standardized and structured recording can, for example, be helpful in determining where to find certain information and organizing clinical reasoning and documentation [25]. In addition, the increased use of coding systems has facilitated the use of routine care data in these settings for secondary purposes.

However, in the long-term care setting, this development is lagging. Although an increasing amount of data are being collected in routine long-term care, for example through a minimal dataset for each patient or health care encounter [26], routine data infrastructure in long-term care remains mostly inadequate for secondary purposes [27]. The use of routine health data from nursing home populations is crucial for both primary use in the (daily) care process and secondary use, for example, in quality improvement and (scientific) research. For primary use, standardized and structured recording in EHRs can help improve data exchange between health care providers. For secondary use, EHR data can help, for example, in scientific research, as traditional research methods such as clinical trials are frequently unsuitable because older adults with complex diseases are often excluded from scientific research [28]. Some initiatives, for example, during the COVID-19 pandemic, have shown that EHR data from nursing homes can be of great value for scientific research [29,30] and policy development [31].

Currently, information in long-term care is mainly recorded in free text and lacks the desired structure and standardization [27]. To improve standardized and structured recording in EHRs in long-term care, it is important to know which factors facilitate or hinder health care professionals. For example, Kharrazi et al [32] mentioned that some geriatric syndromes can be captured in *ICD* or SNOMED CT codes, but many are not well represented in these terminologies. In other health care settings, for example, hospitals, factors such as attitude, subjective norms, institutional trust, perceived risk, perceived usefulness, and perceived ease of use were identified [33,34]. Verheij et al [13] described similar factors that influenced the recording behavior of health care professionals in primary care, including the use of a software system actively adopted by health care providers, the availability of recording guidelines, and the integration of coding systems and thesauruses within the EHR system. Professionals displaying strategic recording behavior because of monetary incentives as well as the influence of awareness of sharing data with other health professionals or patients have also been mentioned by Verheij et al [13]. These factors are all consistent with previously found factors in renowned models of technology acceptance such as the Technology Acceptance Model (TAM) and the Unified Theory of Acceptance and Use of Technology (UTAUT) [35,36]. To the best of our knowledge, no research has been reported on structured and standardized recording in EHRs in long-term care settings. Whether these factors, which are known from previous research in other health care sectors, are also applicable to health care professionals in long-term care should be verified.

In 2020, a national program was launched in the Netherlands called Learning from data (Dutch: Leren van data) to promote uniformity of language and the reuse of routine health care data recorded by elderly care physicians (ECPs) for quality improvement in their own practice [37]. An ECP is a specialized medical practitioner who is responsible for the medical care of nursing home residents, with primary responsibility for treatment [38]. Therefore, the ECP records a comprehensive view of patients’ health in the EHR. This large amount of health data could include valuable information to inform policy, conduct scientific research, and support quality improvement. However, to extract this valuable information, high quality data are required, that are ideally Findable, Accessible, Interoperable, and Reusable (FAIR) [39], and based on structured and standardized recording. Insight into the ECPs’ recording behavior and attitudes toward standardized and structured recording is vital to provide starting points for changing the recording behavior and to be informed about their attitudes toward standardized and structured recording.

### Objectives

This study aimed to answer why and how ECPs record their findings in EHRs and what factors influence them to record in a standardized and structured manner. The findings will be used to formulate recommendations aimed at enhancing standardized and structured data recording in long-term care to promote the reuse of health data for quality improvement and other purposes.

## Methods

### Study Design

A qualitative study design was used in which semistructured interviews were conducted with ECPs. This design facilitates in-depth insights into the complex, context-specific factors shaping ECPs’ recording practices, which are not easily captured through quantitative methods. The semistructured interviews enabled the exploration of key topics while allowing participants to elaborate on their experiences.

### Participants

Interviews were conducted with ECPs and ECPs in training, working in 12 different Dutch nursing home organizations. Because other health care professionals in Dutch nursing homes often work with different EHRs than the ECPs, we decided to interview only ECPs and ECPs in training. Participants were recruited in several ways. First, we reached out to ECPs through various newsletters of 6 Collaborating Academic Elderly Care Networks (Dutch: Samenwerkende Netwerken Ouderenzorg); Gerimedica, the vendor of the EHR system Ysis; and Verenso, the Dutch Association of Elderly Care Physicians. Participants were then invited through the personal networks of the main researcher’s colleagues working in nursing homes. This eventually led to 20 potential participants. After purposive sampling, of the 20 potential participants, 5 (25%) using Ysis were excluded based on the time of application to maintain sufficient variation in the types of EHRs used. In the Netherlands, nursing home organizations can choose which EHR system to implement. The EHR systems most often used by ECPs are Ysis from Gerimedica, Ons from Nedap, Pluriform from Adapcare, Puur from Ecare, and Fierit from Tenzinger [40]. On the basis of expert opinion, we can say that EHR systems are quite heterogeneous and the level of interoperability varies [41]. We strived for diversity in terms of age, gender, health care organization, and use of the EHR system. Of the 15 remaining potential participants, 2 (13%) could not be reached after the initial contact, which eventually resulted in 13 (65%) participants. Data saturation was reached after approximately 10 interviews. Although the last 3 interviews were still conducted as planned, they did not reveal deviating data or additional insights. In total, 13 interviews were conducted.

Participants were contacted through email and received an information letter that included general information about participating in scientific research, the research goal, and how the retrieved data would be handled. In addition, participants were asked to sign an informed consent form before the interview. Before the interview took place, demographic information, such as specific occupation, gender, age, and self-reported level of digital skills, of all participants was collected via email as background information to be able to describe the research participants. The self-reported level of digital skills was estimated using a 5-point scale ranging from 1 (I never use IT) to 5 (I never need help and I find computers and IT quite easy).

### Data Collection

A total of 13 semistructured interviews were conducted between September 2022 and February 2023 by the primary researcher (CAWA). The interviews were guided by a topic list (Multimedia Appendices 1 and 2) inspired by existing literature, including the TAM and UTAUT models [35,36]. The research team (KJJ, MCDB, and MS) contributed to the topic list by reading along as a team of experts with experience in both scientific research and long-term care practice. As a result, the topic list contains knowledge from both daily practice and scientific research in long-term care.

The initial topic list was pilot-tested with 3 ECPs working at the Department of Medicine for Older People at the Amsterdam University Medical Center and adapted for more clarity and to incorporate additional information on factors influencing standardized and structured recording. On the basis of the iterative analysis process, this topic list (Multimedia Appendix 1) was further adapted after 6 interviews to incorporate more in-depth information on attitudes and motives of ECPs regarding standardized and structured recording (Multimedia Appendix 2).

The interviews were conducted face-to-face and were scheduled at a location and time convenient for the participants. Only the interviewer and the participant were present during the interview. All interviews were audio-recorded using the Philips SpeechExec Enterprise Transcribe Voice recorder app [42] and transcribed verbatim. After transcription, all recordings were deleted. The average length of the interviews was 60 minutes.

### Data Analysis

For the analysis of the interviews, we used the inductive thematic analysis method of Braun and Clarke [43]. This method consists of 6 phases. The first phase involved transcribing the interviews and reading them to become familiar with their content. Then, initial codes were generated from the data using open coding. Codes were assigned using MAXQDA 2020 (VERBI Software) [44]. The first 6 interviews were coded independently by 2 researchers (CAWA and YW-J). Discrepancies were discussed until agreement was reached on the coding system. After coding these 6 interviews, the codes were categorized and potential themes were identified. A mind map was used to facilitate this process. All themes were subsequently checked to determine whether the underlying quotes matched the theme. A codebook was created, including descriptions for each theme and subtheme.

This codebook was also used by the main researcher (CAWA) to analyze the remaining 7 interviews, with the possibility of adding codes and themes when necessary. A second researcher (YW-J) checked all coded text segments, and discrepancies were discussed until consensus was reached. The entire process of data analysis was supervised by a senior researcher experienced in the field of qualitative research (MEDB), and themes and subthemes were discussed within the research team.

### Ethical Considerations

The Medical Ethics Committee of VU University Medical Center determined that the Medical Research Involving Human Subjects Act (Dutch: Wet medisch-wetenschappelijk onderzoek met mensen) does not apply to this study and granted a waiver for ethics approval (METc VUmc 2022.0376).

## Results

### Participants

A total of 13 ECPs and ECPs in training who used different types of EHRs were interviewed (Table 1). Most of the participants were female (9/13, 70%), and most of them (9/13, 70%) used the EHR system Ysis, which is the most commonly used EHR system in Dutch nursing homes.

**Table 1:** Demographics of participants (N=13).

Demographics		Participants, n (%)
**Occupation**		
	Elderly care physician	11 (85)
	Elderly care physician in training	2 (15)
**Gender**		
	Men	4 (30)
	Women	9 (70)
	Nonbinary	0 (0)
**Age group (y)**		
	20 to 40	6 (46)
	40 to 60	5 (38)
	60 to 70	2 (16)
**Years of work experience**		
	0 to 10	6 (46)
	10 to 20	4 (30)
	>20	3 (24)
**Years of experience in the current location**		
	0 to 1	4 (30)
	1 to 5	6 (46)
	>10	3 (24)
**Type of EHR^a^**		
	Ysis	9 (70)
	Ons	1 (8)
	Pluriform	2 (14)
	Fierit (formerly Cura)	1 (8)
**Self-reported level of digital skills^b^**		
	1	0 (0)
	2	0 (0)
	3	1 (8)
	4	8 (62)
	5	4 (30)

^a^EHR: electronic health record.

^b^1=“I never use IT”,2=“I sometimes use IT but I struggle a lot using it”, 3=“I can manage but I regularly need help from colleagues”, 4=“I am able to do most things myself but I sometimes need help from colleagues”, and 5=“I never need help and I find computers and IT quite easy to use.”

### Themes

**Figure 1:**
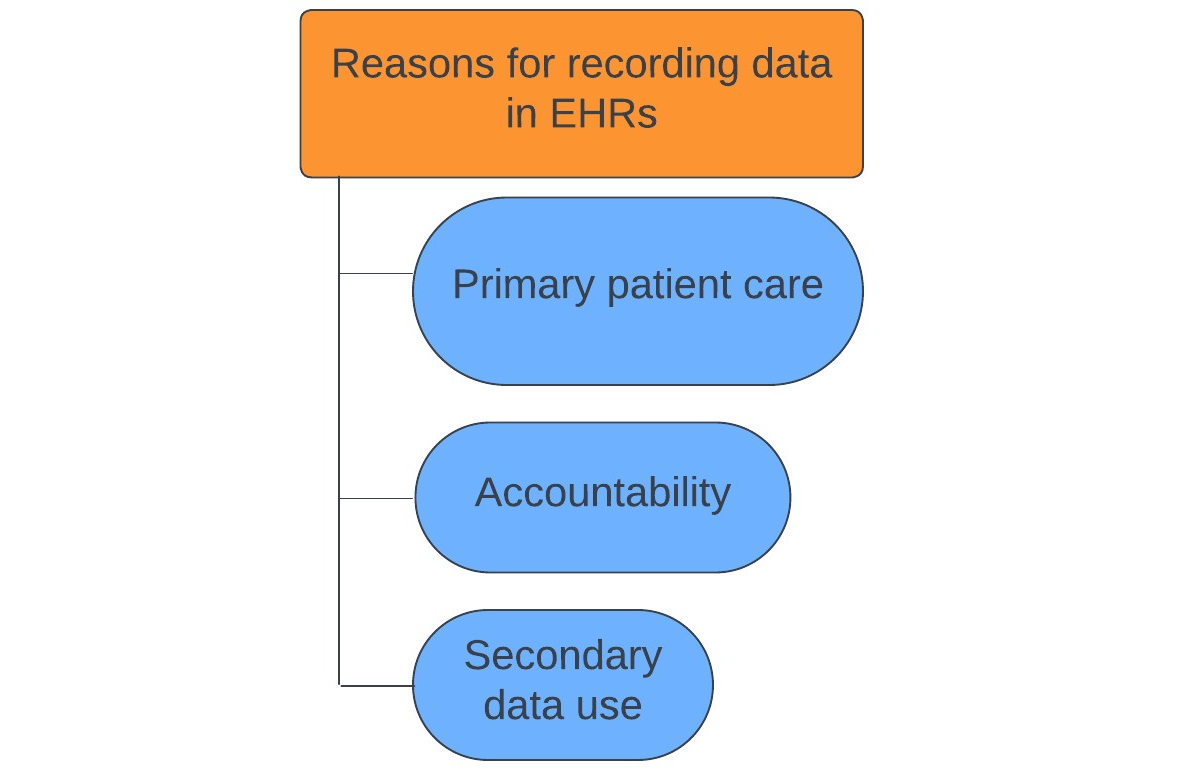
Reasons for recording data in electronic health records (EHRs), including themes.

**Figure 2:**
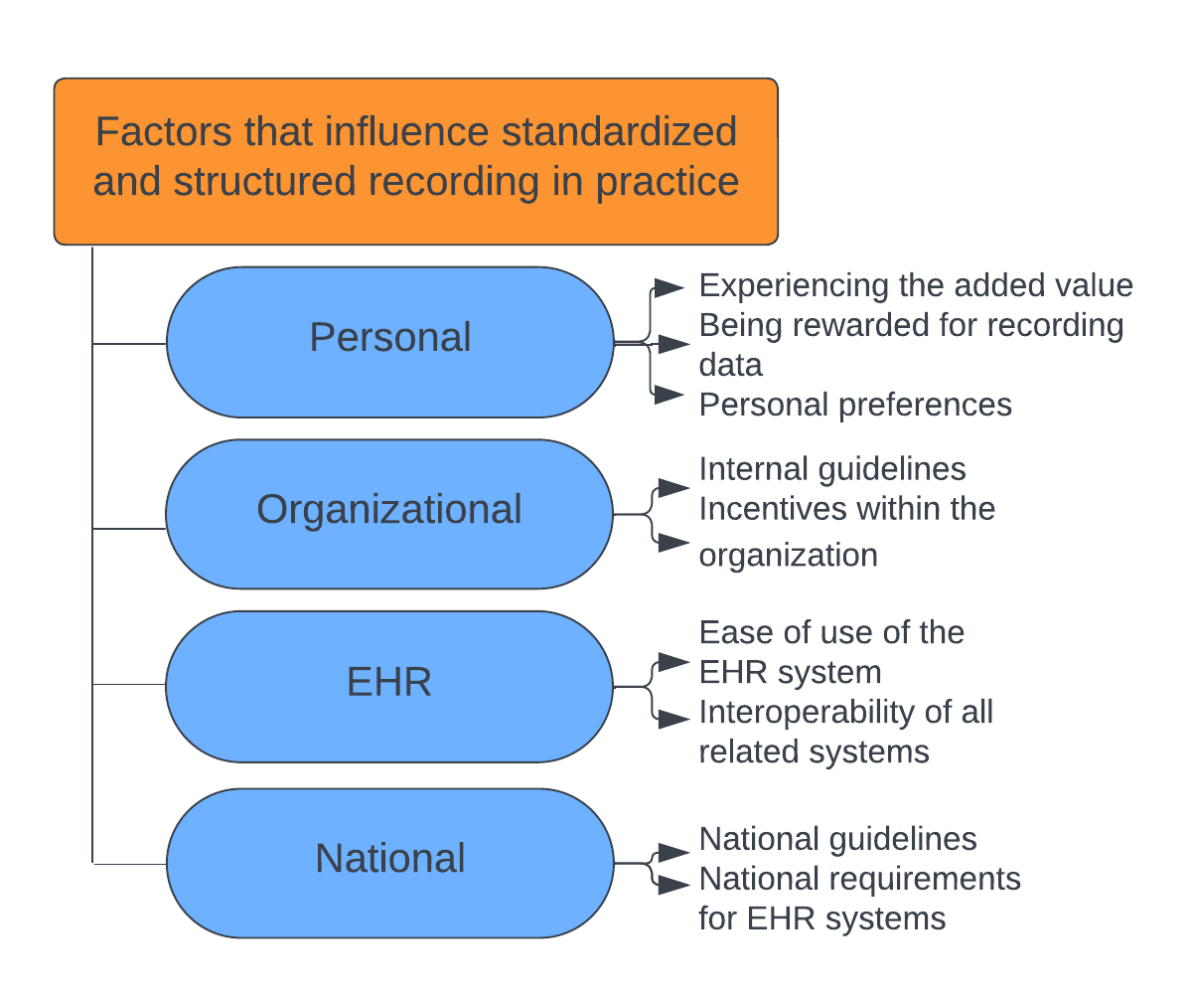
Factors that influence standardized and structured recording in practice, including themes and subthemes. EHR: electronic health record.

Overall, it was found that the terms standardized and structured were not clear and did not mean the same thing to everyone. Therefore, the differences between these ways of recording and recording in general are not always specified in the Results section. In addition, we identified the following themes for both parts of the research question. Regarding the reasons for recording data in EHRs, three themes were identified in order of importance, based on their frequency of mention: (1) primary patient care, (2) accountability, and (3) secondary data use. In addition, four themes were identified as factors influencing standardized and structured recording, organized from the micro to the macro level: (1) personal factors, (2) organizational factors, (3) EHR system–related factors, and (4) national factors. Each of these themes incorporated several subthemes (Figures 1 and 2).

### Reasons for Recording Data in EHRs

#### Primary Patient Care

After the interviews, it became clear that ECPs primarily record health information for primary patient care and continuity of care for their patients. Ensuring continuity of care requires both individual action and communication with others. According to the ECPs, their recordings are an important memory aid for the individual ECP to know about the patient’s treatment and considerations. An ECP stated the following:

...it’s also about being able to read back what your considerations were...

Participant 2, ECP, aged 40-60 y

Besides, it is a way to organize your own ideas and thoughts according to the physicians. The same ECP stated:

...sorting out for yourself, uh, what you’re, so it’s organizing the available data...

Participant 2, ECP, aged 40-60 y

Because of the multidisciplinary nature of long-term care, communication between health care professionals was found to be very important. For ECPs, EHR systems play a critical role in the exchange of health information between health care professionals inside and outside the nursing home. An ECP said:

Yes, for collaboration it’s important, communication.

Participant 4, ECP, aged 20-50 y

These professionals may include general practitioners, medical specialists in the hospital, and pharmacists. Most ECPs considered an effective exchange of information crucial for keeping both immediate colleagues and other health care staff within the organization updated regarding a patient’s status and the course of the treatment they have undergone. In addition, a good exchange of information makes it easier to take over the care of each other’s patients. An ECP commented:

Well, because you could drive into a tree tomorrow...and then your successor needs to know what you’ve done, taking over the care should be no problem...you know?

Participant 5, ECP, aged 40-60 y

Moreover, it is important for ECPs to be able to exchange health information with health care professionals outside the organization when a patient is transferred to and from the nursing home, for example, with general practitioners and medical specialists. Therefore, all physicians indicated that health information exchange through recording in EHRs is crucial for the continuity of care.

#### Accountability

Some ECPs mentioned that they were required to record specific data in EHRs. For physicians in geriatric rehabilitation, for instance, this includes recording time spent per patient to ensure funding. Other ECPs explained their obligation to monitor their patients to ensure and improve the quality of care for the Dutch Health and Youth Inspectorate (Dutch: Inspectie Gezondheidszorg en Jeugd) or to record information in accordance with the Dutch Care and Coercion Act (Dutch: Wet zorg en dwang). Finally, ECPs often mentioned that they record information in EHRs to be able to provide justification in case something goes wrong or in the event of a complaint. An ECP mentioned:

...I think it’s also partly accountability, yes. You do write down a lot just in case.

Participant 9, ECP, aged 40-60 y

#### Secondary Data Use

Another reason for ECPs to record data in EHRs, which ECPs indicated was currently lower on their priority list, was the secondary use of health data for purposes such as scientific research and quality improvement. A tool for quality improvement is for example audit and feedback. A few ECPs mentioned that they see great potential in using already recorded health data for secondary purposes. One of the ECPs stated:

Yes everyone feels scientific research is important.

Participant 3, ECP, aged 20-40 y

However, most ECPs seemed to be primarily concerned with patient care when using EHRs. Another EPC said:

No ... I don’t get the impression that that’s a thing, like oh, we are also reporting for science.

Participant 6, ECP in training, aged 20-40 y

### Factors That Influence Standardized and Structured Recording in Practice

We identified 4 main themes (Figure 2) in the factors that influence standardized and structured recording in practice, which we have arranged from micro to macro level.

#### Personal Factors

##### Experiencing the Added Value

An important intrinsic motivator for recording in a standardized and structured manner was when ECPs experience the added value themselves. An ECP stated the following:

...If I don’t see the relevance of it and I don’t see something in return, not necessarily for me personally, but for the profession or for the development of the target group or whatever. Yes, then I am also willing to invest, as long as I get something in return.

Participant 13, ECP, aged 40-60 y

Few of the participants stated that knowing that data are being recorded but not used can be demoralizing. Some of the interviewees also pointed out the added value for the patient. One of the ECPs said:

It must benefit the patient first and foremost.

Participant 9, ECP, aged 40-60 y

Ultimately, a few ECPs stated that when additional effort is requested to carry out a task, the rationale behind it should be explicitly communicated.

Receiving feedback on recorded information was mentioned by a few ECPs as a possible motivator. One ECP explained:

That was really great for the doctors to get that insight like, so apparently we are a, we have a culture that really is different, or we are doing it exactly right like the guideline...

Participant 1, ECP, aged 20-40 y

Although the added value of reusing health data for scientific research was truly recognized by some ECPs, it was often not something the ECPs believed they could use in their daily practice. According to one ECP:

...you can actually use it for data and for research and, uh, uh, that we thought like, well yes, it’s kind of a nice thing. Well yeah, but in practice we don’t have much use for it anyway.

Participant 10, ECP, aged 20-40 y

##### Being Rewarded for Recording Data

Some ECPs mentioned that being rewarded for recording data could be a motivator to improve their standardized and structured recording. This could be achieved through financial incentives such as pay for performance, or resources such as book vouchers and chocolates to change their recording habits. An ECP stated:

If we get more money for it, I’ll be happy to perform an administrative act.

Participant 10, ECP, aged 20-40 y

By contrast, as mentioned by one of the ECPs, physicians are also expected to contribute to scientific research, and it is questionable whether they should be rewarded for that. An ECP remarked:

I think it’s complicated, because doctors should also contribute to science, an important quality of doctors.

Participant 9, ECP, aged 40-60 y

Some ECPs also said that they would not be motivated to change their recording behavior if they were rewarded for it.

##### Personal Preferences

Most physicians mentioned that they try to record information as uniformly as possible, following their organization’s internal agreements when applicable. However, personal preferences can play a role in how these internal guidelines are applied in practice. In particular, there may be differences in the structured recording in open-text fields, both in terms of where things are recorded and in the degree of detail. Some physicians record more extensively, while others are more concise in their way of recording. One ECP explained:

There is always a little bit of, uh, well, a little bit of local color, so to speak...

Participant 11, ECP, aged 40-60 y

These differences can increase the risk of missing or misinterpreting important information. Standardized recording was also found to be susceptible to personal preferences. ECPs mentioned that they prefer to record information differently when family members of nursing home residents have access to (parts of) EHRs. They record in less detail, use simpler wording, and sometimes even leave things out to avoid unnecessary worry. However, changing personal preferences was found to be difficult. An ECP commented:

Well, it is difficult to adapt your own style to the group, it remains complicated.

Participant 9, ECP, aged 40-60 y

And it is only realistic when the benefits are clear. The same ECP said:

... then we also have to be able to really see the difference. See that we are gaining something from it.

Participant 9, ECP, aged 40-60 y

#### Organizational Factors

##### Internal Guidelines

Some ECPs mentioned that having clear internal guidelines within their organization on how to record in a standardized and structured way can improve uniformity. However, the degree of importance attached to having internal guidelines within the health care organization varies. Several ECPs say that “we actually have a pretty clear agreement with each other,” while others indicate that there is a desire for internal guidelines but that it is currently “not a priority,” for example, because they have just introduced a new EHR system.

##### Incentives Within the Organization

All ECPs indicated that there are multiple ways for an organization to increase the incentives for standardized and structured recording. First, time is an important incentive, which can be enhanced, for example, by reserving time for proper recording, as a lack of time can be a barrier. One of the ECPs explained:

Yes time, a lack of time.... You just look at what has priority.

Participant 3, ECP, aged 20-40 y

Explicitly reserving extra time in, for example, regular meetings to discuss the recording methods could also be useful. An ECP said:

No, the issues of the day leave no time for that [to discuss recording]. So really, an outside person should get themselves invited and that will create space in the physician meetings.

Participant 8, ECP, aged 40-60 y

In addition, it is important to make time for a proper onboarding procedure for new colleagues. An ECP remarked:

So then you have to invest in overlap between doctors, a real familiarization period...

Participant 1, ECP, aged 20-40 y

An adequate staffing level also increases the amount of available time. Second, the organization could place greater emphasis on addressing standardized and structured recording through managers who can remind physicians more often. According to the ECPs, the organization and managers within the organization should create a culture that stimulates alignment with the internal guidelines of the organization, for example, by holding each other accountable for these agreements and by observing what their colleagues do. Third, the organization could play a role in process optimization by transferring straightforward tasks from the physician to, for example, a student or a secretary. Fourth, and very important for some of the ECPs, was having enough tablets and computers to be able to record digitally. Finally, it is suggested that the organization could benefit from having designated super or key users who can assist colleagues when needed and motivate them to record health information in a standardized and structured way.

#### Factors Related to EHR Systems

##### Ease of Use of the EHR System

According to most of the ECPs, the ease of use of the EHR system plays an important role in how much effort it takes to record in a standardized and structured way. Some ECPs state that they prefer to use the preprogrammed structure of EHRs for daily reporting. However, others often look for a more suitable alternative, such as a completely open-text field, if the structure does not match the way they want to record the information. The use of structured fields could be improved in 2 ways. First, ECPs suggested that to increase use, structures should be of additional value to the user, for example, by providing more overview in daily practice. An ECP stated the following:

Well, that does make it more manageable...Yes, so it also kind of forces you to, uh, register in that way. Yes, then it's also easier to retrieve, you know.

Participant 11, ECP, aged 40-60 y

Second, some of the ECPs indicated that the EHR system could guide the users toward a more standardized and structured way of recording, for example, by increasing the number of mandatory fields that must be filled in before a form can be completed. An ECP commented:

In my experience not being able to go forward if you don't fill it in works very well.

Participant 9, ECP, aged 40-60 y

ECPs mentioned that drop-down menus could be added to limit the number of options that can be filled in a particular field. Finally, some also mentioned limiting the possibility of recording information in free-text fields, for example, by making the free-text fields less visible or by removing them altogether.

Another important factor concerning the ease of use of the EHR system for standardized recording is the link between a classification system for diagnoses, such as the *ICD-10*, and practice. Although some ECPs use classification systems to record health information in EHRs, most ECPs indicated that they make limited use of standardized codes for diagnoses because they do not match what they want to report. One of the ECPs indicated:

Then you have this picture in your head of what it is, but that just doesn’t quite fit into the ICD-10 coding.

Participant 7, ECP, aged 20-40 y

Furthermore, the addition of *ICD* or *ICPC* codes to patients’ medical histories is often deliberately avoided. An ECP stated:

We all work around it. Because it’s much too time-consuming to enter each one separately.

Participant 3, ECP, aged 20-40 y

Most ECPs pointed out that they prefer to use “descriptive diagnoses,” which was confirmed by one of them saying, “in our profession, I think it’s really important that you are able to describe the nuances” because long-term care involves a generalist profession. Therefore, ECPs often add information in free-text notes to accompany a diagnosis.

##### Interoperability of All Related Systems

The ECPs did not mention interoperability—the ability of different information and communication technology systems and software applications to communicate; to exchange data accurately, effectively, and consistently; and to use the information that has been exchanged—as a commonly used term [45]. However, most of them did say that they consider it important that all systems, such as systems for prescribing medication, receiving laboratory results, and general practitioner and hospital data, are interoperable. Interoperability implies interdependency; according to the respondents, interoperability will also lead to improved recording, as interoperability works best when patient information is recorded in a standardized and structured way.

Interoperability between the EHR systems used by ECPs and systems used by other health care professionals is found to be very important. Even within a nursing home organization, different systems may be operational. In such cases, transferring patient health records to nurses and other care staff who use an EHR system that is different from the ECP’s is perceived as time consuming. As one ECP explained:

Then you can’t copy all the text in one go and save it to the care file or the client’s record, so then you have to copy everything four times.

Participant 7, ECP, aged 20-40 y

Moreover, interoperability with systems outside the nursing home, such as general practice systems, is a valuable motivator for improved standardized and structured recording. One of the current problems mentioned is the transfer of patient files from the general practice or hospital system to the nursing home EHR system, or vice versa. This is still very difficult and time consuming. An ECP stated the following:

It’s like a daily battle for data.

Participant 9, ECP, aged 40-60 y

Because patient files are still often exchanged as PDF files, there is no incentive to use the standardized and structured recording method. The drug prescription system or the laboratory management system is another system that should be interoperable with the EHR system. ECPs stated that when this process works smoothly, it results in high-quality care. As stated by one of the ECPs:

So the best thing is that the current medication, when you open a letter, can also be entered into [EHR name], so there is communication...that’s high quality I think. That’s excellent.

Participant 1, ECP, aged 20-40 y

Some ECPs mentioned that when all EHR systems communicate well enough, recordings in certain places of the EHR are automatically copied to other EHR systems. Consequently, this is a motivation to record patient data in a standardized and structured format.

In addition to interoperability with existing systems, a few ECPs expressed hopes for recording support from artificial intelligence. Artificial intelligence could, for example, help ECPs create standardized formats from free text or suggest diagnoses based on symptoms.

#### National Factors

##### National Guidelines

Although there are national guidelines on how to record in EHRs for general practitioners [46] and medical specialists [47], there is no guideline for ECPs made by, for example, the professional association. Some ECPs indicated that they would be open to committing to these guidelines. An ECP indicated the following:

If they [the professional association] say about that the professional guidelines from Verenso are very valuable in the nursing home. Great importance is attached to that.

Participant 9, ECP, aged 40-60 y

As mentioned by some of the ECPs, having national guidelines could be of great help not only for organizations but also for medical specialty training. ECPs suggested that recording agreements in the medical specialty training should be based on these national guidelines. According to the ECPs, this could lead to more national uniformity in how to use EHRs and how to record, and this could ultimately lead to a better foundation for young physicians.

##### National Requirements for EHR Systems

According to some of the ECPs, stricter national regulations and legislation regarding the structure of the EHR system could increase the uniformity of recording in health record systems and could decrease the influence of the EHR vendor. One of the interviewees mentioned the importance of these regulations. The ECP stated:

...it’s all about the free market, right? I think putting very strict requirements on your ECD. Yes, legislating that...

Participant 4, ECP, aged 20-40 y

ECPs mentioned that these national requirements could oblige EHR vendors to create more uniformity in their EHR systems and to give more direction within the EHR system toward standardized and structured recording following the national guidelines.

## Discussion

### Principal Findings

This study aimed to answer why and how ECPs record their findings in EHRs and what factors influence them to record in a standardized and structured manner. ECPs were found to have 3 main reasons for recording data in EHRs. First, they used these data for day-to-day patient care and to ensure continuity of care. Second, ECPs are required to record specific information for accountability reasons and to be able to justify themselves in the event of a complaint. Finally, ECPs recorded data in EHRs for the secondary use of data; this is currently less of a priority than other reasons. We found considerable variation among ECPs in the extent to which they record information in a standardized and structured manner within EHRs. We identified 4 factors that influence this behavior: personal, organizational, EHR system–related, and national factors.

Our finding that ECPs mainly record data in EHRs for primary patient care and to be able to guarantee continuity of care is in line with other research in the hospital setting where medical specialists state that “the primary goal of healthcare is to treat the patient, and that reuse of data is therefore no incentive” [13,48]. We found similar factors that influence standardized and structured recording, such as organizational policies, national policy, structure of and support from the EHR system, support in the recording process, (monetary) incentives, sharing data, and knowledge and time [13,21].

An important factor in standardized and structured recording is having a well-functioning EHR system within the organization. In addition, according to Verheij et al [13], the functionalities available within EHR systems may affect the completeness, correctness, and precision of recorded data. Differences in the functionalities of EHR systems can also influence recording methods. We found that the EHR system should be easy to work with and the functionalities of the EHR system should support standardized and structured recording. Furthermore, different systems used by various users should be interoperable to reduce the additional work involved in using EHRs in practice. In addition, the coding systems and thesauruses integrated into EHR systems determine what can be recorded in a standardized manner [32], thereby influencing semantic interoperability. Improving semantic interoperability in health care offers benefits such as more usable data for improved patient care, higher quality of care, and the ability to use clinical data for other purposes [19]. Our findings show that in long-term care, these systems and thesauruses still do not align with daily practice, and the codes are often not in line with the diagnoses that ECPs have in mind. Therefore, it is of great importance that the coding function in EHR systems is in line with the daily practice of ECPs to improve semantic interoperability.

In addition to a well-functioning EHR system, there are some incentives to motivate ECPs to record in a standardized and structured way. Our findings suggest that returning information about the data, for example, through audit and feedback, can be a motivator to improve the way of recording. This is supported by previous research conducted in hospitals [49]. Research in general practice also has shown that a data quality feedback tool substantially improves standardized recording in EHRs [50]. In addition, Klappe et al [21] found that structured recording in EHRs can be enhanced by highlighting direct benefits, such as saving time and effort, or by reusing standardized lists for consultation notes or letters to motivate physicians in training.

Another way to encourage standardized and structured recording is through (monetary) incentives. The so-called pay for performance, that is, receiving money to provide care or record certain information, can improve the quality of care and is a commonly used resource to improve physicians’ recording. Although pay for performance seems to be effective in improving the quality of recording in general practice [51,52] and large integrated health care delivery systems [53], it remains questionable whether this would also work in the nursing home setting. Our research shows a difference of opinion among ECPs on being rewarded for standardized and structured recording of data; while some ECPs said they were open to it, others expressed doubts about its effectiveness.

Finally, our results demonstrate the importance of national and internal guidelines on how to record data in EHRs. If national guidelines were developed, for example, by professional associations, they could be used as a basis for developing internal guidelines. Research in hospital care has also found that guidelines explaining how to use specific parts of the EHR system should be created and shared with staff [25] and introduced during peer-to-peer training sessions [48]. In addition to national guidelines, international rules and legislation can promote standardized and structured recording in EHRs. A relevant initiative in this context is the European Health Data Space (EHDS), which aims to improve interoperability and develop reporting guidelines [54]. Standardized and structured recording is essential to the EHDS’s goal of facilitating health care data exchange. It may also require EHR vendors to make their electronic health systems more compatible and uniform in recording formats. In addition, increased data availability across Europe could also encourage physicians to adopt standardized and structured recording. Our study supports the EHDS’s goal by providing insights into the factors improving standardized and structured recording in nursing homes to enhance (semantic) interoperability. Future research could explore how uniformity in EHR systems and recording practices affect data quality in nursing homes.

Existing technology acceptance models, such as TAM and UTAUT [35,36], which cover the domains of performance expectancy, effort expectancy, social influence, and facilitating conditions, as well as the model proposed by Joukes et al [34] for the adoption of structured and standardized data recording among health care professionals confirm the factors identified in our study, such as attitudes, environmental factors, and the quality of the (data from the) EHR system. They also provided relevant recommendations for tailored strategies to improve current recording behavior. One recommendation could be to improve physicians’ knowledge and skills in recording in a standardized and structured way. We also suggest communicating the potential benefits of standardized and structured recording and how it can improve work processes. It is important to pay more attention to standardized and structured recording within the organization. In addition, organizations should have a proper onboarding procedure, reliable computers and devices, and key users who can assist and motivate colleagues in adopting standardized and structured recording practices. Finally, the opinion of the users of EHR systems regarding the quality of the system should be improved to create trust in the system’s functionalities. Given that we found results similar to previous studies in other health care sectors, one implication is that long-term care could learn from primary and hospital care to improve standardized and structured recording. Furthermore, because of the important social component in long-term care [55], we could also examine the recording process in the social domain and how broad social perspectives are incorporated in their standardized and structured recordings.

### Strengths and Limitations

A key strength of our research is that we are the first in the field of long-term care to investigate standardized and structured recording by ECPs in EHRs, as previous research has focused primarily on the implementation of EHR systems in nursing homes [56,57]. Moreover, our study benefited from the use of semistructured interviews, which allowed respondents to share what was most important to them and provided a comprehensive perspective on the use of EHR systems and information recording practices. In addition, keeping in mind the applicability of our results to the Dutch nursing home context, we carefully recruited a representative group of respondents. Finally, we explored the full scope of the research topic because we reached data saturation. A limitation of this study is that we did not examine the differences in opinions and experiences between users of different EHR systems. For example, there may be differences in how EHR systems look and work, and this may influence how easy standardized and structured recording is and how ECPs feel about it. Another limitation is that we cannot rule out socially desirable responses, which may have led to a slight bias toward more positive behavior. Finally, our interviews were limited to physicians (ECPs and ECPs in training), but it would also be valuable to gather the perspectives of other health care professionals who use the same EHR systems. Therefore, future research could focus on including other target groups such as other medical practitioners and nursing specialists.

### Conclusions

Our findings are similar to those of previous studies in hospital care and general practice. Therefore, long-term care can learn from solutions regarding standardized and structured recording in other health care sectors. Primary use in daily patient care and continuity of care are the main motives for ECPs to record data in EHRs. The key leverage point for promoting standardized and structured recording lies in the primary care process, which can be achieved in roughly 2 ways. First, standardized and structured recording can be improved by aligning the method of recording data in EHRs with the primary process. Second, there are incentives to motivate ECPs to record in a standardized and structured way. Incentives exist at the personal, organizational, EHR system, and national levels.

## Appendix

Multimedia Appendix 1 First topic list.

Multimedia Appendix 2 Second topic list.

## Abbreviations

ECP: elderly care physician
EHDS: European Health Data Space
EHR: electronic health record
ICD: International Classification of Diseases
ICPC: International Classification of Primary Care
SNOMED CT: Systematized Nomenclature of Medicine Clinical Terms
TAM: Technology Acceptance Model
UTAUT: Unified Theory of Acceptance and Use of Technology

## Data Availability

The datasets generated and analyzed during this study are not publicly available due to confidentiality reasons but are available in anonymized form from the corresponding author on reasonable request.
