# Supplementary material for: Enhancing Standardized and Structured Recording by Elderly Care Physicians for Reusing Electronic Health Record Data: Interview Study
Source: JMIR Med Inform. 2024 Dec 13;12:e63710. doi: 10.2196/63710 (PMC11681280; doi:10.2196/63710)
Supplement: Multimedia Appendix 1 [file medinform_v12i1e63710_app1.docx]

**Multimedia Appendix 1**

Topic list 1 - Interviews with elderly care physicians

Components of the interview:

1. Introduction

2. What does standardized and structured recording mean?

3. Why do ECPs record data and what do they consider important?

4. Facilitators: what is going well and what causes this?

5. Barriers: what makes standardized and structured recording not go well?

6. How can we improve the current level/manner of recording?

**Introduction**

- Since when have you been an ECP? Did you previously work in another specialism?
- Apart from your work as an ECP, are you also active in other specialties? E.g., in an advisory role, on a board, committee, in the professional association, etc.?
- Did you receive any training or courses on working with the EDP beforehand? How particular things should be recorded or which rules to follow?
- Is there a training document for new employees? If so, what does it look like?
- Are there any experts/colleagues within your institution that you can turn to with questions about using the EHR?

**What does standardized and structured recording mean?**

- Are you familiar with one or both terms?
- Are you applying standardized and structured recording in your work?
- What do you think that the goals of standardized and structured recording are?
- Are you aware of the Minimal Data Set (MDS) and do you include it in the patient record? If not, why not?

**Why do ECPs record data and what do they consider important concerning this?**

- Can you explain why you record data in EHRs?
- What are your goals in recording data?
- What do you expect others to minimally record or how should certain things be recorded?
- Can you explain a little bit more about whether and which data you record where?

o Medical history (how extensive/limited)

o Physical examination

o ADL functioning

o Medical history

o Policy

o Medication

o Family interviews

o Ordering of the daily recordings: problem-oriented or time-oriented?

- Are there guidelines on how to record? Does every colleague record in the same way? If so, how do you learn that? What does it mean if nothing is recorded?
- Are there certain requirements that recordings in EHRs must meet? These could be national or within your institution. Are you required to record in a certain way?

**Facilitators: what is going well and what causes this?**

- Are there particular factors that make recording easier?
- Are there particular factors that encourage you to record?
- How do your colleagues feel about the use of EHRs and standardized/structured recording? In your opinion, does this differ between institutions?
- Does every ECP record in the same way? What are the differences?
- How can we promote uniformity in structure?

**Barriers: what makes standardized and structured recording not go well?**

- In your opinion, are there factors that make you less likely to record in a standardized and structured way?
- Is it easy to use EHRs? If yes/no, why yes/no?
- Research shows that recording is still far from being done in a standardized way. For example, much information is entered in the free text fields instead of in the correct text fields. Do you recognize this? What could be the reason for it?

**How can we improve the current level/manner of recording?**

- What would make standardized and structured recording easier?
- What do you think is the benefit of standardized and structured recording?
- Do you already use data from EHRs for purposes other than direct patient care? E.g., Data returned from the medication prescription system during a Pharmacotherapeutic Consultation
- How is this information returned?
- Suppose we want to use data from EHRs for purposes other than patient care. Think of quality improvement, policy development or scientific research. What would that require? What is your view on this? And what purposes would you have in mind?
- Do you work with coding systems such as ICD or ICPC?
