# Supplementary material for: Enhancing Standardized and Structured Recording by Elderly Care Physicians for Reusing Electronic Health Record Data: Interview Study
Source: JMIR Med Inform. 2024 Dec 13;12:e63710. doi: 10.2196/63710 (PMC11681280; doi:10.2196/63710)
Supplement: Multimedia Appendix 2 [file medinform_v12i1e63710_app2.docx]

**Multimedia Appendix 2**

Topic list 2 - Interviews with elderly care physicians

Brief introduction of the topic and purpose of the interview. Then briefly introduction to each other.

Purpose of the interview: find out whether there is support among ECPs for more standardized and structured recording for data reuse and find out what would be needed for this.

**Introduction**

- What are you currently doing concerning recording? Can you take me along some steps?
- What do you think is important in this?
- What do you struggle with?
- What does it mean for your daily work?
- Own goals to record?
- Do you work with coding systems such as ICD or ICPC?
- Do you use recording guidelines?

**Experiences**

- We would like to start recording increasingly standardized and structured, such as in hospitals and general practice, for the reuse of data for multiple purposes. What do you think about that?
- Could it be of additional value?
- Is that desirable?
- And if so, in what way?
- What conditions should it meet?

**Suggestions**

- ECP: how did you learn to record or how do you teach others to record in a certain way?
- Organization: onboarding program? EHR system training? Is there enough attention for?
- Agreements within the organization or professional group?
- Time: factor for good recording?
- Money: factor? Could promote recording?
- EHR system: user-friendly?
- Concerns about data privacy/security?
- Support in using the EHR system?
- Interoperability with other systems?
- National policy?

**Conclusion**

- Give summary and name important conclusions!
- Is there anything else you would like to share?
- Did we miss important parts?
